# Dose-Dependent Effects of Acute Caffeine Ingestion on Physical and Cognitive Performance in Trained Female Handball Players: A Randomized Crossover Study

**DOI:** 10.3390/life16060954

**Published:** 2026-06-05

**Authors:** Murat Turgut, Ulas Can Yildirim, Akan Bayrakdar, Aydan Ermis, Idris Kayantas, Selin Yildirim Tuncer, Izzet Karakulak, Mehmet Can Gundem, Deema Mohammed Alogaiel, Monira I. Aldhahi

**Affiliations:** 1 Department of Sport Management, Faculty of Sport Sciences, Sinop University, Osmaniye 57100, Türkiye; mturgut@sinop.edu.tr (M.T.); mcgundem@sinop.edu.tr (M.C.G.); 2 Department of Coaching Education, Faculty of Sport Sciences, Sinop University, Osmaniye 57100, Türkiye; ucyildirim@sinop.edu.tr; 3 Department of Coaching Education, Faculty of Sport Sciences, Alanya Alaaddin Keykubat University, Alanya 07400, Türkiye; akan.bayrakdar@alanya.edu.tr; 4 Department of Foreign Languages, School of Foreign Languages, Ondokuz Mayıs University, Atakum 55200, Türkiye; aydanak@omu.edu.tr; 5 Department of Coaching Education, Faculty of Sport Sciences, Bingol University, Central 12000, Türkiye; ikayantas@bingol.edu.tr; 6 Department of Coaching Education, Faculty of Sport Sciences, Lokman Hekim University, Çankaya 06510, Türkiye; selin.yildirim@lokmanhekim.edu.tr; 7 Department of Sport Management, Faculty of Sport Sciences, Mardin Artuklu University, Artuklu 47100, Türkiye; izzetkarakulak@artuklu.edu.tr; 8 Health Sciences Department, College of Health and Rehabilitation Sciences, Princess Nourah bint Abdulrahman University, P.O. Box 84428, Riyadh 11671, Saudi Arabia; dmalokaiel@pnu.edu.sa; 9 Department of Rehabilitation Sciences, College of Health and Rehabilitation Sciences, Princess Nourah bint Abdulrahman University, P.O. Box 84428, Riyadh 11671, Saudi Arabia

**Keywords:** caffeine, dose response, physical performance, cognitive performance, handball players

## Abstract

Handball requires athletes to sustain intermittent high-intensity effort while maintaining rapid cognitive processing and technical skills. Caffeine is widely used as an ergogenic aid, yet its dose-dependent effects across physical, cognitive, and technical performance outcomes remain unclear in female handball players. This randomized, double-blind, placebo-controlled crossover study examined the acute effects of low-dose caffeine (LCAF; 3 mg/kg), moderate-dose caffeine (MCAF; 6 mg/kg), and placebo (PLA) in trained female handball players. Participants (*n* = 20) completed three experimental sessions separated by 72 h. Cognitive performance was assessed using the Simplified Eriksen Flanker Test, throwing performance was evaluated through maximal ball velocity, and intermittent running capacity was measured with the Yo-Yo Intermittent Recovery Test Level 1. Both LCAF and MCAF significantly improved Yo-Yo performance compared with PLA (*η^2^p* = 0.415, representing improvements of approximately ~23.5% and 29.0%), with no difference between caffeine doses. MCAF significantly reduced overall Flanker response time (*η^2^p* = 0.486, ~18.5%) and congruent and incongruent trial response time compared with PLA and LCAF. No significant effects were observed for throwing velocity, Flanker accuracy and interference scores. These findings suggest that acute caffeine intake has performance-specific effects in female handball players: intermittent running performance responded to both doses, whereas cognitive enhancement was limited to response time, with no improvements in inhibitory control or accuracy.

## 1. Introduction

Team sports are characterized by a dynamic structure where high-intensity physical effort and complex cognitive processes, such as rapid decision-making, awareness, and inhibitory control, interact alongside technical skills [1]. Optimal performance requires the development of cognitive performance, such as attention and decision-making, in addition to physical capabilities [2]. Especially under conditions of fatigue, competition over limited neurocognitive resources can directly and negatively impact the application of technical skills and decision-making processes. This impact becomes particularly critical in sports like handball, which require multiple skills such as explosive movements, accurate shots, and quick decision-making under defensive pressure and fatigue [3]. Especially during the later stages of a game, when physiological and mental fatigue accumulate, the ability to maintain technical accuracy and cognitive efficiency becomes a key determinant of performance outcomes [4,5]. The studies in the literature show that increased physical and mental load disrupts attention resources, leading to decreased decision-making quality and increased technical errors [6,7]. This highlights the need for ergogenic strategies that can simultaneously support both physical and cognitive performance components, rather than approaches that only improve isolated performance capacities.

Caffeine, whose primary mechanism of action is adenosine receptor antagonism, is widely accepted to stimulate the central nervous system, increase neurotransmitter and beta-endorphin release, and increase plasma catecholamine levels and plasma renin activation [8,9]. These mechanisms underlie its well-known ergogenic effects, such as endurance capacity, neuromuscular performance, and reaction time [10,11]. However, the magnitude of these physiological and ergogenic responses is subject to considerable individual variability. This variability primarily depends on genetic differences in the CYP1A2 enzyme in the liver. CYP1A2 enzyme determines the clearance rate of caffeine and its conversion into active metabolites such as paraxanthine [12], thus influencing individual tolerance and optimal dosing strategies. Caffeine is frequently used as a potential performance enhancer. Indeed, improvements in endurance performance and the ability to sustain high-intensity exercise during simulated team sports activities have been reported in the literature [8,13]. From a cognitive and sport-specific technical skills perspective, caffeine has been shown to improve attention and alertness, particularly under conditions of fatigue. However, its effects on higher-level cognitive processes such as control and decision-making appear to be more variable and task-dependent [14,15]. While one study reported improvements in pass accuracy and decision-making performance in soccer players [16], Field et al. reported contrasting results, including improvements in reaction time under fatigue but impairments in decision-making performance [15]. This difference can likely be attributed to a speed–accuracy imbalance resulting from increased central nervous system stimulation. While caffeine effectively speeds up simple neuromotor processing and reaction time, overstimulation or increased impulsivity can compromise higher-level executive functions and perceptual-motor sensitivity necessary for complex and dynamic decision-making [17]. Furthermore, several studies and reviews indicate that caffeine may have negligible or context-dependent effects on repeated sprint performance and specific sport skills and cognition [8,18,19]. All these findings suggest that the ergogenic effects of caffeine are not homogeneous and depend on factors such as exercise type, task sequence and complexity, and specific performance outcomes.

While a significant part of research on caffeine supplementation focuses on physical performance outcomes [19,20], a more limited literature has examined its effects on cognitive performance [16,21]. In the literature, evidence exists but is limited for studies that simultaneously assess physical and cognitive outcomes within the same experimental protocol in individual and team sports where multiple motor skills, technical proficiency, and decision-making processes determine performance [22,23,24]. This limitation is even more pronounced in the case of trained female handball players, as the majority of historical data relies on male cohorts, overlooking potential metabolic variations such as differences in caffeine clearance inherent to female physiology. Furthermore, findings regarding the effects of commonly used caffeine doses in sports performance studies, such as 3 and 6 mg/kg, are inconsistent, and these doses need to be evaluated not only in terms of performance outcomes but also in terms of individual tolerance and dose optimization [8,25]. In particular, whether different doses have similar or different effects on the same performance parameters is important for the practical impact of research. In this context, holistically addressing the effects of two commonly used caffeine doses on physical and cognitive performance in trained female handball players can be considered a novelty in the research.

This randomized, double-blind, placebo-controlled crossover study compared low caffeine (LCAF; 3 mg/kg), moderate caffeine (MCAF; 6 mg/kg), and placebo conditions to evaluate their effects on intermittent running performance, throwing ability, and cognitive performance. The aim of this study is to examine the dose-dependent effects of caffeine on trained female handball players. Although the literature reports context-dependent and sometimes contradictory results regarding cognitive tasks, caffeine’s broad physiological mechanisms provide a strong theoretical basis for its comprehensive ergogenic effects. In particular, its simultaneous roles in neuromuscular activation, accelerating central nervous system neurotransmission, and delaying both central and peripheral fatigue demonstrate its potential for performance enhancement. Therefore, it was hypothesized that LCAF and MCAF conditions would improve all performance parameters compared to placebo.

## 2. Materials and Methods

### 2.1. Participants

**Figure 1:**
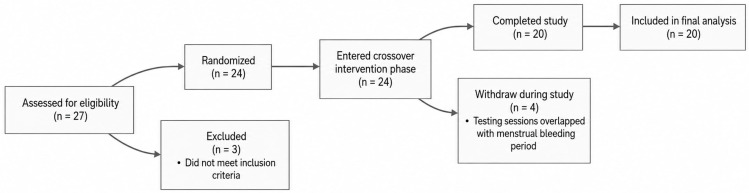
Consort Flowchart.

The required sample size was determined using G*Power software (version 3.1.9.4; Düsseldorf, Germany) based on an analysis of variance (ANOVA) design including repeated measures and within-subject factors [26]. The calculation was based on the overall experimental design rather than on a domain-specific cognitive or physical outcome, because both cognitive and physical performance outcomes were assessed under the same within-subject supplementation structure and analyzed using the same repeated-measures model. For the analysis, a within-subject correlation of *r* = 0.85 was assumed. An effect size (*f*) of 0.25, a significance level (alpha) of 0.05, and a statistical power of 0.90 were used. As a result, 20 trained female handball players (19.85 ± 1.98 years, 165.3 ± 6.37 cm height, 61.48 ± 10.15 kg body mass, 22.51 ± 2.98 kg/m^2^ BMI and 4.2 ± 1.5 years of training experience) participated in this study (Figure 1).

To improve transparency, we additionally examined how the achieved power would change under more conservative assumptions for the correlation among repeated measures, while keeping the other G*Power parameters unchanged. When this correlation was set at *r* = 0.60, the achieved power was 0.75. When it was further reduced to *r* = 0.50, the achieved power was 0.65. These values show that the available sample size was mainly supported by the original assumption of high within-subject stability. Under more conservative field-test assumptions, the study had lower power to detect small or more variable effects. For this reason, non-significant findings should be interpreted with caution.

Participants’ body composition measurements were performed during the first laboratory visit. Height was measured using a Seca stadiometer (Seca Deutschland, Hamburg, Germany), while body fat percentage was assessed using an InBody 770 body composition analyzer (InBody Co., Gangnam-gu, Seoul, Korea). Inclusion criteria were: (i) being a female handball player aged 18–25, (ii) being trained, and (iii) not having sustained a serious musculoskeletal injury in the past six months. Also, eligibility for participation was restricted by specific exclusion criteria. Exclusion criteria included: (i) caffeine sensitivity or allergy, (ii) chronic use of supplements or medications affecting performance, and (iii) any cardiovascular or metabolic disorders. To minimize the potential influence of menstrual-cycle-related hormonal variation on physical and cognitive performance, experimental sessions were scheduled according to each participant’s self-reported menstrual cycle. Testing sessions were planned outside the menstrual bleeding period for all participants (i.e., targeting the late follicular or luteal phases). Four participants were excluded because their scheduled testing sessions overlapped with the menstrual bleeding period (Figure 1). Additionally, none of the participants reported using oral contraceptives. The study was approved by the Sinop University Human Research Ethics Committee (2025/688), and all participants provided written informed consent prior to enrollment in accordance with the Declaration of Helsinki.

### 2.2. Study Design

This study was conducted using a randomized, double-blind, placebo-controlled crossover design to evaluate the acute effects of different caffeine doses on physical and cognitive performance in trained female handball players. Participants completed a familiarization, followed by three experimental (LCAF; 3 mg/kg body mass, MCAF; 6 mg/kg body mass and PLA; placebo) conditions, with a 72 h washout period between sessions to minimize potential carryover effects. All conditions were prepared in identical, opaque and red capsules containing either polydextrose or anhydrous caffeine and were ingested 60 min prior to testing by participants. The sequence of the three experimental conditions was determined using a computer-generated Latin square design. Both participants and researchers were blinded to the experimental conditions.

**Figure 2:**
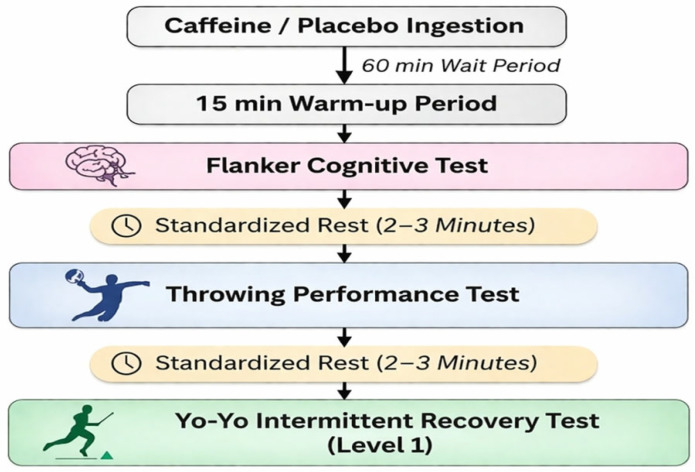
Test Protocol.

Participants followed a standard 15 min warm-up protocol consisting of 5 min of low-intensity running (approximately 50% of maximum heart rate), followed by 5 min of dynamic stretching, and finally 5 min of handball-specific movements (passing, jumping, and shooting). Following the warm-up protocol, the tests consisted of Flanker cognitive test, maximal ball velocity test and Yo-Yo Intermittent Recovery Test Level 1 (Figure 2). Standardized rest intervals (2–3 min) were given between tests for recovery.

### 2.3. Data Collection Tools

All physical and cognitive assessments were conducted under standardized indoor conditions. The cognitive test was performed in a quiet environment using a laptop computer and standard wireless mouse, while physical performance tests were carried out in an indoor sports hall on a standard handball court. Environmental conditions (temperature: 22–24 °C; relative humidity: 50–60%), including lighting and ambient noise, were kept consistent across all sessions. Testing sessions were conducted at the same time of day (09:00–10:00) to minimize potential circadian influences on performance. The sequence of tests was standardized as follows: (i) Simplified Eriksen Cognitive Test, (ii) maximal ball velocity test and (iii) Yo-Yo Intermittent Recovery Test Level 1. Standardized rest times were provided between tests to minimize fatigue and carryover effects.

#### 2.3.1. Simplified Eriksen Flanker Cognitive Test

The Simplified Eriksen Flanker Test (CogniFit, Inc., New York, NY, USA, version 2024.1) was used to assess inhibitory control and selective attention, which are essential components of rapid decision-making. This test is a computerized neurocognitive assessment tool based on the original Eriksen-Flanker task [27]. Participants viewed a horizontal array of five arrows and were instructed to identify the direction of the central target arrow by pressing the corresponding left or right button, while ignoring the surrounding arrows, which could be either congruent or incongruent with the target. The test comprised a familiarization (4 randomized trials; 2 congruent, 2 incongruent) and a testing phase (24 randomized trials; 12 congruent, 12 incongruent). Stimuli were presented centrally and responses were limited to 5000 milliseconds. Primary outcomes consisted of mean accuracy (%), response time (milliseconds; correct trials only) overall, by condition, omission errors (no-response trials), and flanker effects (incongruent minus congruent).

#### 2.3.2. Maximal Ball Velocity Test

Throwing performance was assessed to evaluate maximum ball velocity, a critical performance indicator closely related to scoring ability in handball. From the standard 7 m line, all players performed a standing throw with their dominant hand toward a standard handball goal, using an International Handball Federation (IHF) approved size 2 handball. Three maximal effort trials were completed, with 30 s of standardized rest between trials to control for fatigue accumulation. All trials were measured using a Stalker Solo 2 radar gun (model ATS, Plano, TX, USA) positioned behind the goal at a 3 m distance. Velocities were recorded in km/h, and the mean of the three trials was calculated and used for statistical analysis.

#### 2.3.3. Yo-Yo Intermittent Recovery Test—Level 1 (Yo-Yo IRT-1)

Participants’ intermittent aerobic endurance capacity was assessed with Yo-Yo Intermittent Recovery Test Level 1. This test is widely used in the literature to evaluate field performance in team sports with intermittent high-intensity running profiles, such as handball [28]. Participants performed 2 × 20 m shuttle runs at progressively increasing speeds controlled by pre-recorded audio beeps, with 10 s active recovery consisting of 2 × 5 m jogging between shuttles. All players began each shuttle run from behind the 20 m line, reaching the far line and returning before the subsequent beep. Speed increases according to the standard Yo-Yo IR1 audio protocol. The test terminated upon two failures to reach the finish line in time or voluntary exhaustion. The total distance was recorded (m) as the primary performance outcome.

### 2.4. Supplement Protocol

To ensure blinding, visually identical red capsules were used in all supplement conditions. The supplementation conditions were as follows: (i) placebo (PLA), consisting of polydextrose; (ii) 3 mg/kg anhydrous caffeine (LCAF) and (iii) 6 mg/kg anhydrous caffeine (MCAF). The doses were prepared using a precision laboratory balance (Shimadzu, Tokyo, Japan). Oxford pure caffeine powder (ISO 14001−98.5% purity; The Oxford Vitality Health Company Ltd.; London, UK) and polydextrose (Litesse Ultra, Danisco USA Inc., Terre Haute, IN, USA) were used throughout the study.

### 2.5. Diet and Caffeine Consumption Control

Participants were instructed to abstain from alcohol consumption and intense physical activity for at least 24 h prior to each session. Throughout the study period, participants were asked to refrain from using any additional dietary supplements. To ensure consistency in dietary intake, participants recorded their food consumption during the 24 h preceding all sessions. These records were reviewed by the research team to confirm consistency in overall energy intake and macronutrient composition across sessions.

Habitual caffeine intake was assessed with a survey [29] before the first session (173.77 ± 64.15 mg/day). To minimize potential caffeine withdrawal effects, participants were instructed to maintain their habitual caffeine consumption throughout the study period.

### 2.6. Statistical Analyses

Statistical analyses were performed using IBM SPSS Statistics (version 25.0, IBM Corp., Armonk, NY, USA), and graphical visualizations were generated with GraphPad Prism (version 10.0; GraphPad Software, San Diego, CA, USA). Data normality was assessed using the Shapiro–Wilk test for each outcome under all experimental conditions. Sphericity was evaluated with Mauchly’s test, and Greenhouse–Geisser corrections were applied when violations occurred. Potential order effects were examined prior to the main analyses using repeated-measures ANOVA, including supplementation order as a factor. No significant period or order effects were observed (*p* > 0.05), indicating that the washout period (72 h) was sufficient to minimize potential carryover effects.

Outcomes were analyzed using one-way repeated-measures ANOVA to evaluate the effects of supplementation condition (PLA, LCAF, MCAF). When significant main effects were detected, Bonferroni-adjusted pairwise comparisons were conducted. Secondary Flanker analyses examining response time and accuracy in congruent and incongruent trials were performed only when significant effects were observed in the primary analyses. Effect sizes were reported as partial eta squared (*η^2^p*) (small, ≥0.01; moderate, ≥0.06; or large, ≥0.14), and pairwise comparisons were additionally expressed as Cohen’s *d* (trivial, <0.20; small, 0.20–0.49; moderate, 0.50–0.79; or large, ≥0.80) for repeated measures [30]. Statistical significance was set at *p* < 0.05. All data are presented as mean ± standard deviation (SD).

## 3. Results

A significant effect of supplementation condition was observed for Yo-Yo Intermittent Recovery Test Level 1 performance (*F* = 13.49, *p* < 0.001, *η^2^p* = 0.415). Participants covered greater distances following both LCAF and MCAF compared with PLA, while no difference was observed between the two caffeine doses. Descriptive results showed that performance increased from 964 ± 277 m under the placebo condition to 1191 ± 383 m with LCAF and 1244 ± 396 m with MCAF. Post hoc comparisons indicated that both LCAF (*p* < 0.001) and MCAF (*p* < 0.001) significantly improved Yo-Yo performance compared with placebo, while no significant difference was observed between LCAF and MCAF (Table 1).

No significant main effect of supplementation condition was detected for throwing velocity (*F* = 0.04, *p* = 0.961, *η^2^p* = 0.004). Mean throwing velocity remained similar across conditions (PLA: 73.79 ± 6.05 km/h; LCAF: 73.95 ± 6.49 km/h; MCAF: 73.71 ± 6.60 km/h), indicating that acute caffeine ingestion did not influence maximal ball velocity in female handball players (Table 1).

A significant main effect of supplementation condition was observed for Flanker response time (*F* = 17.95, *p* < 0.001, *η^2^p* = 0.486). Reaction time progressively decreased across supplementation conditions, with the fastest responses observed in the MCAF condition (601.95 ± 135.48 ms) compared with LCAF (716.30 ± 107.59 ms) and PLA (738.50 ± 122.62 ms). Post hoc analyses revealed that MCAF significantly reduced response time compared with both PLA (*p* < 0.001) and LCAF (*p* < 0.001), whereas the difference between PLA and LCAF was not statistically significant (*p* = 0.97). Because a significant main effect was detected for overall response time, additional analyses were conducted separately for congruent and incongruent trials. A similar pattern was observed for congruent trials, with response times of 707.36 ± 146.12 ms for PLA, 703.77 ± 109.87 ms for LCAF, and 590.79 ± 134.70 ms for MCAF (*F* = 11.20, *p* < 0.001, *η^2^p* = 0.371). For incongruent trials, response times were higher across all conditions but followed the same trend (*F* = 10.64, *p* < 0.001, *η^2^p* = 0.542) (Table 2).

Pairwise comparisons indicated higher Yo-Yo IRT-1 distances after both LCAF and MCAF compared with PLA. The mean difference was 227.00 m for LCAF versus PLA (95% CI: 88.55 to 365.45) and 280.00 m for MCAF versus PLA (95% CI: 110.04 to 449.96). The comparison between MCAF and LCAF showed no clear difference (53.00 m, 95% CI: −87.58 to 193.58). Throwing velocity was similar across conditions, with mean differences of 0.16 km/h for LCAF versus PLA (95% CI: −1.82 to 2.15), −0.08 km/h for MCAF versus PLA (95% CI: −1.96 to 1.80), and −0.24 km/h for MCAF versus LCAF (95% CI: −2.48 to 1.99). For cognitive outcomes, MCAF was associated with shorter response times than both PLA and LCAF. Overall response time was lower in MCAF compared with PLA (−136.56 ms, 95% CI: −209.02 to −64.09) and LCAF (−114.35 ms, 95% CI: −176.23 to −52.48), while the LCAF–PLA comparison was not clearly different (−22.21 ms, 95% CI: −79.57 to 35.16). The same pattern was observed for congruent trials: MCAF versus PLA, −116.57 ms (95% CI: −194.62 to −38.53); MCAF versus LCAF, −112.98 ms (95% CI: −181.46 to −44.51); and LCAF versus PLA, −3.59 ms (95% CI: −77.40 to 70.22). For incongruent trials, response time was also lower in MCAF compared with PLA (−157.86 ms, 95% CI: −249.10 to −66.63) and LCAF (−116.71 ms, 95% CI: −193.27 to −40.15), whereas LCAF did not clearly differ from PLA (−41.16 ms, 95% CI: −112.07 to 29.75). Accuracy showed minimal between-condition differences: 0.00% for LCAF versus PLA (95% CI: −0.79 to 0.79), −0.21% for MCAF versus PLA (95% CI: −1.17 to 0.76), and −0.21% for MCAF versus LCAF (95% CI: −1.17 to 0.76) (Table 3).

**Figure 3:**
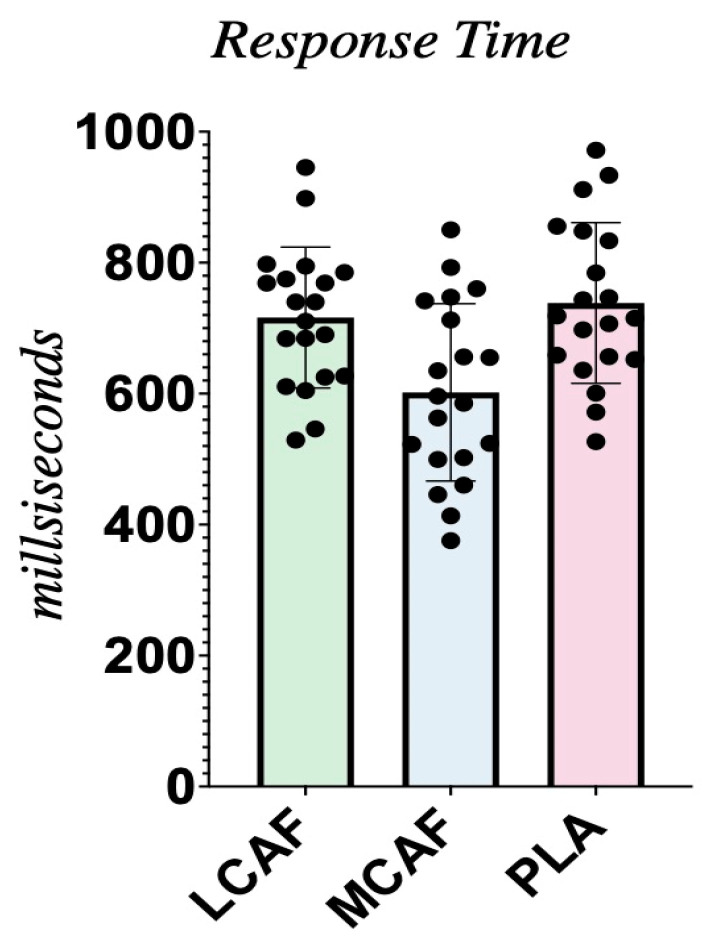
Simplified Eriksen Flanker response time across conditions.

Despite these reductions in response time (Figure 3), no significant main effect of supplementation condition was observed for Flanker interference (flanker effect) (*F* = 1.01, *p* = 0.37, *η^2^p* = 0.051) or Flanker accuracy (*F* = 0.24, *p* = 0.79, *η^2^p* = 0.013).

## 4. Discussion

The findings of this study indicate that acute moderate-dose caffeine intake reduced reaction time in the Simplified Flanker task in trained female handball players (~18.5%), whereas the lower dose produced limited and non-significant effects. In addition, both low and moderate doses of acute caffeine intake were seen to increase Yo-Yo IRT-1 performance compared to placebo (~23.5% and 29.0% respectively). However, there was no difference between the conditions in the throwing velocity parameter and flanker task accuracy, and interference effects were similarly unchanged by supplementation. Importantly, these findings show that the effects of acute caffeine on performance differ across performance parameters. While physical and cognitive outcomes are interrelated performance components, they do not respond uniformly to ergogenic interventions such as caffeine.

A significant effect was observed in particular on the general response time (*F* = 17.95, *p* < 0.001, *η^2^p* = 0.486). Post hoc analyses showed that the response time in the MCAF condition was significantly reduced compared to both PLA and LCAF conditions. A similar result was detected in congruent trials (*F* = 11.20, *p* < 0.001, *η^2^p* = 0.371) and incongruent trials (*F* = 10.64, *p* < 0.001, *η^2^p* = 0.542). This decrease in reaction time after moderate-dose caffeine is consistent with the effects of the cognitive processing speed of caffeine [17,31]. This effect can largely be explained by caffeine antagonizing adenosine receptors. The reduction of adenosine’s inhibitory effect increases central nervous system excitability [32,33], while the increase in the release of catecholamines such as dopamine and norepinephrine can support alertness, focus, and neural processing efficiency [34]. However, these mechanistic explanations remain speculative as direct physiological and biochemical measurements were not performed in the present study. This finding, which may contribute to a reduction in central fatigue perception, is consistent with the broader literature demonstrating caffeine’s effectiveness in positively regulating psychophysiological responses and perceived exertion in high-intensity individual and team sports [35,36,37]. This mechanistic explanation is consistent with previous research that caffeine may support cognitive performance in sports tasks that require reaction time, rapid attention shift, and short-term decision-making [14,30]. Similarly, Bello et al. reported that applications containing caffeine under simulated match conditions in high-level footballers may have positive effects on cognitive performance, especially selection response time [13]. On the other hand, the fact that there was no significant difference between low-dose caffeine and placebo suggests that this effect may be due to a specific dose threshold [38]. These findings suggest that, while low-dose caffeine has a limited stimulating effect on cognitive performance, moderate stimulation may be required to significantly support rapid information processing and psychomotor reactivity in team sports.

Despite these marked reductions in overall, congruent and incongruent reaction times, the lack of significant change in Flanker accuracy and interference effect warrants careful interpretation (accuracy: *F* = 0.24, *p* = 0.79, *η^2^p* = 0.013; Flanker effect: *F* = 1.01, *p* = 0.37, *η^2^p* = 0.051). In tasks where high accuracy is typically emphasized, such as the Flanker cognitive test, elite or well-trained athletes may exhibit ceiling effects that limit the potential for further improvement in accuracy. The fact that accuracy values are at the ceiling level (~99%) in all conditions, along with high participant motivation, may also mean that this particular cognitive task is not demanding enough to sufficiently challenge the neurocognitive resources of trained athletes [13,16]. Therefore, the observed reduction in reaction time without corresponding changes in interference scores likely reflects the aforementioned overall increase in central nervous system arousal and alertness, rather than a true increase in higher levels of executive inhibitory control. These results support the trend that caffeine facilitates faster responses without sacrificing accuracy for speed [16,39,40]. Furthermore, recent evidence highlights that psychomotor and cognitive responses to exercise and ergogenic aids can be significantly modulated by individual factors, including gender and daily variations [41,42].

Both low and moderate-dose acute caffeine intake improved Yo-Yo Intermittent Recovery Test Level 1 performance compared to placebo, but no significant difference was observed between doses (*F* = 13.49, *p* < 0.001, *η^2^p* = 0.415). The current findings are consistent with previous studies reporting potential ergogenic effects of caffeine on high-intensity interval running performance in team sports. While López-Samanes, et al. [43] reported an increase in running capacity in futsal players, Ranchordas, et al. [44]. reported that repeated sprint performance in football players improved after caffeine intake. In addition, Bello, et al. [13] revealed that practices containing caffeine under simulated match conditions can have positive effects on exercise tolerance and match-like endurance performance in high-level footballers. When these studies are evaluated together, it reinforces the interpretation that caffeine can support intermittent high-intensity performance specific to team sports. In contrast to cognitive outcomes, the absence of dose-dependent differences between LCAF and MCAF suggests that intermittent endurance performance may reach a plateau at relatively lower caffeine doses. A similar result has been reported by Yildirim [45], who observed that both low (3 mg/kg) and moderate (6 mg/kg) doses of caffeine improved aerobic endurance in trained female handball players without significant differences between doses. This divergence between cognitive and physical responses reinforces the notion that ergogenic effects should be interpreted within a multi-dimensional performance framework rather than as uniform enhancements across all domains.

No significant difference in maximal ball velocity was observed among all conditions (*F* = 0.04, *p* = 0.961, *η^2^p* = 0.004). This finding can be explained by the fact that handball throwing speed depends not only on strength or power production but also on neuromuscular specificity, technical proficiency, coordination, and timing of the multi-segmental kinematic chain. Ball throwing in handball is a complex motor skill requiring a high level of coordination of force transferred from the lower extremities to the trunk, shoulder girdle, and upper extremity. Therefore, caffeine’s potential to increase central nervous system stimulation, alertness, or general activation levels may not directly translate to the fine motor control, muscle activation sequence, and technical calibration processes that determine throw speed. While caffeine can improve high-intensity outputs such as sprinting and jumping, its effects may not directly translate to complex sport-specific skills like handball throw, where technical execution and intersegmental coordination are key determinants of performance [16,43,44]. Also, since throwing technique is often optimized near the individual performance ceiling, especially in trained athletes, the potential for achieving additional ergogenic gains solely through acute neurophysiological stimulation may be limited. Moreover, in this study, evaluating throwing velocity with a static 7 m standing throw is a closed skill task. While methodologically reliable, it does not fully reflect the perceptual-motor complexity, dynamic constraints, and decision-making elements specific to real handball movements under game conditions. It is conceivable that a more ecologically sound protocol, such as a jump throw performed under time pressure, defensive interference, or physiological fatigue, might yield different results by better reflecting the multifaceted demands of the sport. Similar findings have been reported by Yildirim [45], where caffeine improved aerobic endurance but not throwing velocity in trained female handball players. A similar effect was observed in soccer players, where caffeine ingestion did not improve ball-kicking speed in trained players [46].

Several limitations should be acknowledged for this research. (i) The relatively small sample size may limit statistical power and generalizability. Specifically, our power analysis assumed a high within-subject correlation (*r* = 0.85) based on general test–retest reliabilities; however, if the actual correlation between the intervention conditions is more conservative, the study may be underpowered to detect smaller effect sizes. (ii) Inclusion of only female handball players restricts the applicability of the findings to other populations. (iii) The acute supplementation design captures short-term responses but does not reflect chronic use. (iv) Additionally, the use of a single cognitive assessment limits the scope of executive function-related outcomes and generalization, particularly given the observed ceiling effect in accuracy, which suggests the task may not have been sufficiently demanding for this trained group. (v) Also, evaluating maximal ball velocity with static 7 m standing shots lacks the necessary ecological validity to fully reflect the perceptual-motor complexity of match conditions. (vi) Furthermore, while brief Flanker-based assessments are possible in settings where repeated measurements are applied, the relatively small number of trials conducted may have reduced the sensitivity and reliability of the Flanker result, particularly in detecting subtle changes in inhibitory control. (vii) Objective physiological parameters such as heart rate, blood lactate, and plasma caffeine concentration were not evaluated in this study. This limited our ability to validate the specific mechanisms driving the observed responses. (viii) Lastly, to maintain ecological validity and reduce the risk of caffeine withdrawal symptoms, participants were allowed to continue their usual daily caffeine intake before testing. Therefore, the experimental caffeine doses were taken in addition to their habitual caffeine consumption. The lack of a standardized pre-test abstinence period, such as avoiding caffeine-containing products for 12–24 h before each session, is a potential limitation of the study. Since habitual caffeine intake was not strictly controlled before each visit, baseline caffeine status may have differed between participants and, possibly, between sessions. This may have influenced individual responses to the experimental doses, including a possible weakening of the observed effect of the lower dose or a stronger apparent response to the moderate dose in some athletes. Future research should examine a broader range of cognitive tasks, different caffeine doses, including lower or higher dose, and timing strategies, including sport-specific decision-making paradigms. Studies including mixed-gender groups and varying levels of competition will enhance generalizability. Furthermore, examining the interaction between physical and cognitive demands under conditions of fatigue may provide a more ecologically valid understanding of performance in team sports settings.

## 5. Conclusions

In conclusion, this study demonstrates that acute caffeine intake produces dose-dependent effects on some parameters in trained female handball players. Both low (3 mg/kg) and moderate (6 mg/kg) doses of caffeine provided a clear ergogenic benefit, significantly improving intermittent running performance test results, which are consistent with the nature of handball. In contrast, only the moderate (6 mg/kg) dose increased cognitive response speed. Lastly, neither dose affected cognitive inhibitory accuracy nor throwing velocity. These findings suggest that caffeine does not have a similar effect on all performance components. The magnitude of the effect appears to depend on the physiological load of the relevant task and technical-decision-making requirements. While caffeine can provide a more pronounced advantage in rapid information processing and psychomotor reactivity, its direct transfer to motor skills that require a high level of coordination and technical application may be limited. Given the small sample size and limited design of this study, practical recommendations regarding caffeine dosage should be applied with caution. While our data suggest that moderate doses may support cognitive processing speed under pressure and low doses may be sufficient for intermittent endurance, these findings need further validation in larger populations and longitudinal contexts before definitive strategies can be formulated. Athletes and practitioners should tailor caffeine use to individual response and specific performance requirements, but also bear in mind that its effects on technical proficiency appear to be limited. Moreover, practitioners must consider that higher caffeine doses (e.g., 6 mg/kg) may induce adverse side effects, such as gastrointestinal distress, anxiety, or insomnia, particularly during late-day activities. Therefore, it is highly recommended that coaches and athletes systematically try this dosage in controlled training environments to monitor individual tolerance before implementing it in official competitions.

## Figures and Tables

**Table 1:** Physical performance outcomes and one-way repeated-measures ANOVA statistics.

Parameters	PLA (Mean ± SD)	LCAF (Mean ± SD)	MCAF (Mean ± SD)	*F*	*p*	*η^2^p*
Yo-Yo IRT-1 (m)	964 ± 277	1191 ± 383	1244 ± 396	13.49	0.001 *	0.415
Throwing velocity (km/h)	73.79 ± 6.05	73.95 ± 6.49	73.71 ± 6.60	0.04	0.961	0.004

* *p* < 0.05.

**Table 2:** Cognitive performance outcomes and one-way repeated-measures ANOVA statistics.

Parameters	PLA (Mean ± SD)	LCAF (Mean ± SD)	MCAF (Mean ± SD)	*F*	*p*	*η^2^p*
Response Time (ms)	738.50 ± 122.62	716.30 ± 107.59	601.95 ± 135.48	17.95	0.001 *	0.486
Congruent Trial (ms)	707.36 ± 146.12	703.77 ± 109.87	590.79 ± 134.70	11.20	0.001 *	0.371
Incongruent Trial (ms)	770.46 ± 119.20	729.30 ± 126.87	612.59 ± 150.61	10.64	0.001 *	0.542
Flanker effect (ms)	33.10 ± 105.25	25.53 ± 101.08	21.81 ± 89.73	1.01	0.37	0.051
Accuracy (%)	99.79 ± 0.93	99.79 ± 0.93	99.58 ± 1.28	0.24	0.79	0.013

* *p* < 0.05.

**Table 3:** Pairwise comparisons and 95% confidence intervals for conditions.

Parameters	LCAF vs. PLA Mean Diff. (95% CI)	MCAF vs. PLA Mean Diff. (95% CI)	MCAF vs. LCAF Mean Diff. (95% CI)
Yo-Yo IRT-1 (m)	227.00 (88.55, 365.45)	280.00 (110.04, 449.96)	53.00 (−87.58, 193.58)
Throwing velocity (km/h)	0.16 (−1.82, 2.15)	−0.08 (−1.96, 1.80)	−0.24 (−2.48, 1.99)
Response Time (ms)	−22.21 (−79.57, 35.16)	−136.56 (−209.02, −64.09)	−114.35 (−176.23, −52.48)
Congruent Trial (ms)	−3.59 (−77.40, 70.22)	−116.57 (−194.62, −38.53)	−112.98 (−181.46, −44.51)
Incongruent Trial (ms)	−41.16 (−112.07, 29.75)	−157.86 (−249.10, −66.63)	−116.71 (−193.27, −40.15)
Accuracy (%)	0.00 (−0.79, 0.79)	−0.21 (−1.17, 0.76)	−0.21 (−1.17, 0.76)

## Data Availability

The original contributions presented in the study are included in the article; further inquiries can be directed to the corresponding author.
